# Creating High-Resolution Multiscale Maps of Human Tissue Using Multi-beam SEM

**DOI:** 10.1371/journal.pcbi.1005217

**Published:** 2016-11-21

**Authors:** André F. Pereira, Daniel J. Hageman, Tomasz Garbowski, Christof Riedesel, Ulf Knothe, Dirk Zeidler, Melissa L. Knothe Tate

**Affiliations:** 1 Graduate School of Biomedical Engineering, University of New South Wales, Sydney, Australia; 2 Carl Zeiss Microscopy GmbH, Oberkochen, Germany; 3 Orthopaedic and Rheumatologic Institute, Cleveland Clinic, Cleveland, Ohio, United States of America; 4 TissuTex Pty. Ltd., Wentworth Falls, New South Wales, Australia; The Johns Hopkins University School of Medicine, UNITED STATES

## Abstract

Multi-beam scanning electron microscopy (mSEM) enables high-throughput, nano-resolution imaging of macroscopic tissue samples, providing an unprecedented means for structure-function characterization of biological tissues and their cellular inhabitants, seamlessly across multiple length scales. Here we describe computational methods to reconstruct and navigate a multitude of high-resolution mSEM images of the human hip. We calculated cross-correlation shift vectors between overlapping images and used a mass-spring-damper model for optimal global registration. We utilized the Google Maps API to create an interactive map and provide open access to our reconstructed mSEM datasets to both the public and scientific communities via our website www.mechbio.org. The nano- to macro-scale map reveals the tissue’s biological and material constituents. Living inhabitants of the hip bone (*e*.*g*. osteocytes) are visible in their local extracellular matrix milieu (comprising collagen and mineral) and embedded in bone’s structural tissue architecture, *i*.*e*. the osteonal structures in which layers of mineralized tissue are organized in lamellae around a central blood vessel. Multi-beam SEM and our presented methodology enable an unprecedented, comprehensive understanding of health and disease from the molecular to organ length scale.

## Author Summary

Until recently, the assessment of organ and tissue health relied on site-sampling (biopsy) of micro-scale regions and was fraught with sampling errors. Overcoming these limitations requires a means for seamless imaging of organs, from their cellular inhabitants to whole organs, akin to charting a map of the organ and its resident cells. Map navigation necessitates the capacity to zoom in and out of regions of interest, with high precision, as well as to analyze relationships between cells, tissue degeneration and organ (patho-)physiology. Here we describe the process, in technical detail, based on a world-first case study of a human hip sample and its resident cell population. We acquired 55,000 nm-resolution images of the hip using multi-beam scanning electron microscopy (mSEM). To reconstruct the entire dataset, we developed stitching algorithms to maximize map precision at smallest length scales, and rendered them using the Google Maps API. This enabled the exploration of the hip and its inhabitants in a seamless manner, from a global to a high-resolution local view of a single cell. The resulting navigable maps are available for research teams and the public alike to explore and to elucidate the cellular basis of tissue degeneration and organ failure (mechbio.org).

## Introduction

Cells and cellular connectivity undoubtedly play a substantial role in tissue and organ scale behavior, yet mechanisms by which higher order system characteristics emerge from local cellular and molecular phenomena remain a conundrum. Recent advances in coupled, multiscale imaging and modeling of biological systems promise to transform the fields of physiology and medicine [1,2]. Within that context, bridging across length scales presents a fundamental challenge [3–5]. Until recently, this challenge was addressed by studying large, complex systems using imaging modalities with increasing resolution, linking the macroscopic and nanoscopic worlds. This approach resulted in single, specific fields of interest with increasing resolution but also with the intrinsic risk of sampling error, which could lead to devastating consequences for a variety of medical applications (*e*.*g*. biopsy). The previous lack of a suitable imaging approach that enables a seamless rendering of organ to subcellular structures provided the impetus to apply multi-beam scanning electron microscopy (mSEM), a rapid throughput, high-resolution technology originally developed for quality control of silicon wafers [6].

Electron microscopy (EM) can resolve morphological details at the nanometer scale and is commonly used to characterize the structural and functional properties of biomaterials, biological tissues, and their cellular inhabitants [7,8]. The acquisition speed of EM, however, limits the capturing of high-resolution images within reasonable time frames and therefore typically is limited to areas within the micrometer range [9]. In contrast, multi-beam scanning electron microscopy (mSEM) circumvents typical throughput limitations inherent to conventional single-beam scanning electron microscopes (sSEM) [6]. Its novel design enables the analysis of nanoscale morphologies across macroscopic specimens by implementing parallel electron beams and a multi-channel detector [10,11]. Multi-beam SEM is capable of reducing acquisition time by more than one order of magnitude and, therefore, of imaging larger surface areas with remarkable resolution [11], paving the path for seamless multiscale imaging of organ systems down to the cellular and even molecular scale [12]. As a consequence, this technology has drawn interest within the scientific community, particularly in areas related to brain connectomics [13,14] and cross-scale musculoskeletal mechanobiology [2,10]. Already, a few recent studies in connectomics have utilized mSEM to render volumetric image data from murine specimens and reconstruct neuronal circuits with single-synapse resolution [13,14]. Another recent study from our lab demonstrated the feasibility of using high resolution, navigable multiscale maps of human tissue, created for the first time with mSEM, to assess organ- to cell-scale health using epidemiological approaches [12]. Here we describe technical challenges and solutions for the creation of such maps for biomedical applications, using human sample datasets obtained with a mSEM prototype.

**Fig 1:**
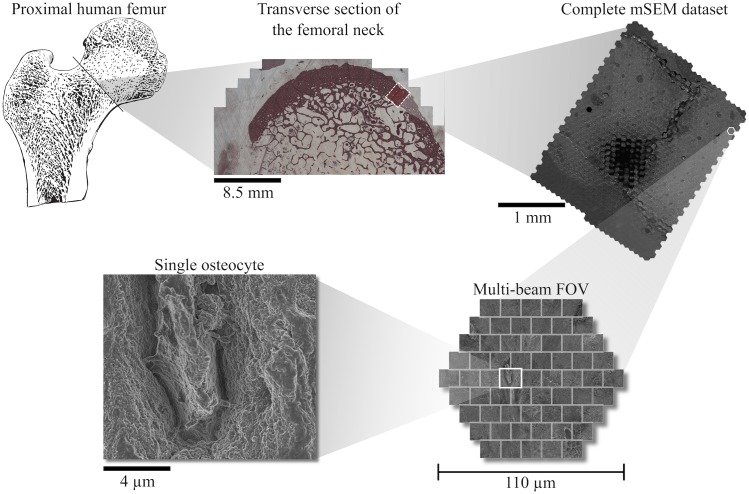
Raw image data organization. Femoral neck tissue samples were acquired from human patients undergoing hip replacement. An area of approximately 2.4 x 2.4 mm was imaged with a 61-parallel-beam Zeiss MultiSEM 505 prototype. The entire dataset was composed of 897 multi-beam fields-of-view, each comprised of 61 tiles with a resolution of 10 nm.

Current multi-beam SEM acquisitions run at speeds up to 1.2 MPixel/s, and typical acquisitions often result in terabyte-size datasets comprised of thousands of individual image tiles that, once combined, form one large complete image (Fig 1). Initial coordinates of individual image tiles are recorded by the microscope stage. However, the precision of the stage can be worse than that of the nanometer resolution electron microscope. Additionally, the interactions between electron beams and specimen samples, as well as residual illumination aberrations exacerbated when imaging organ samples containing large, dense tissue areas, e.g. bone, affect the relative positions of beams during mSEM imaging. These effects lead to tile misalignment, which can then be addressed by a process commonly referred to as *image stitching*. Stitching algorithms align and reconstruct sets of overlapping image tiles into seamless photomosaics and are widely used in a variety of fields including microscopy, contemporary digital mapping and panoramic photography. However, upon implementation of stitching and compilation of the overarching image, another challenge remains that is the management, analysis, and dissemination of large data repositories necessary to harness the power intrinsic to mSEM in multiscale characterization of materials.

Following the extraction of preliminary images of human hip tissue using an mSEM prototype, we developed a novel computational framework for the reconstruction of mSEM datasets. Stitching was accomplished by combining image pixel-based alignment with global registration accomplished using motion dynamics of mechanical systems. Using the Google Maps API, we created an interactive map of our dataset. Our computational framework potentiates the power of mSEM to enable seamless, multiscale study of organ systems comprised of tissues and their cellular inhabitants. Here we describe this computational framework, its inherent challenges, as well as potential directions for the future.

## Methods

### 2.0. Ethics Statement

Human hip bone samples were collected by Dr Ulf Knothe, of the Orthopaedic and Rheumatologic Institute of Cleveland Clinic, in accordance with Institutional Review Board protocol #12–335. This protocol involved collection of tissues normally discarded in the course of surgery. Due to this and the anonymization of all tissue samples prior to processing for specimen preparation and imaging, as well as later reporting of data, no consent was necessary.

### 2.1. Specimen Acquisition

Femoral neck tissue samples were acquired from human patients (age and gender not disclosed) undergoing hip replacement and prepared according to techniques adapted from atomic force microscopy studies [15]. To facilitate chemical fixation, these samples were sectioned along the coronal and transverse planes, respectively. All specimen acquisitions were completed by the Department of Cleveland Clinic Surgical and Pathology units, per IRB protocol guidelines [16].

### 2.2. Specimen Preparation

Undecalcified tissues were fixed in 2.5% glutaraldehyde, 4% formaldehyde, and 0.2M cacodylate buffer at 4°C. These tissues were then processed for bulk embedding in poly(methyl methacrylate) (PMMA) to promote gradual polymerization within a vacuum environment. Upon polymerization of the embedding medium, the specimens were polished, or precision CNC-milled, to achieve mirror-like planarity. Thereafter, samples were prepared for carbon coating and imaging. Selective etching took place, between imaging steps, using 0.02M HCl for 90s and/or 10% NaOCl for 11 min, per our previous atomic force microscopy protocols [15,17]. This enabled imaging of the respective organic or inorganic phase of the extracellular matrix from correlating tissues of the hip joint complex.

### 2.3. Multi-SEM Imaging

One human sample was imaged with a 61-parallel-beam Zeiss MultiSEM 505 prototype, which operates with parallel electron beams arranged hexagonally to minimize electron-optical aberrations [10], using a landing energy in the range of 1–3 keV, 100 ns of dwell time per beam, and a resolution of 10 nm. A surface area spanning 5.7 mm^2^ was imaged, resulting in 897 hexagonally shaped multi-beam fields of view (mFOV), comprised of nearly 55 thousand high-resolution image tiles and a total of 75 thousand megapixels (Table 1). Each mFOV was composed of 61 rectangular, single-beam image tiles arranged in a flat, hexagonal pattern (Fig 1), with a frame size of 1288 x 1120 pixels for each tile. Tile overlap ranged from 2.4–55%. The stage used for this study operated with a precision of 2 μm. Image files were stored as bitmap files, accumulating circa 77 GB of storage space. Pixel coordinates for individual single-beam images were available from the microscope metadata, providing a first approximation for relative positioning.

**Table 1:** Dataset information.

Approximate scanned area (mm^2^)	5.69
Number of mFOVs	897
Total number of tiles	54,717
Number of pixels (megapixels)	75,276
Combined image dimensions (pixels)	318,848 x 352,895
Number of image pair overlaps	180,136
MB/mFOV	88.2
Total GB	77.35

### 2.4. Image Alignment and Stitching

A Fourier-based direct alignment algorithm with a simple 2D planar motion model was considered to estimate the translational offsets between overlapping images [18]. Libraries from the registration toolkit TrakEM2 (Fiji) [19,20] were employed to calculate phase correlations [21] for each pair of overlapping image tiles, extracting a metric of pixel-to-pixel similarities, the correlation coefficients, and translation vectors that maximize pairwise alignment quality. The correlation coefficient and 2D translation vectors for the alignment of tiles *i* and *j* are here denoted, respectively, as *R*^*ij*^ and **p**^*ij*^.

Due to the lower quality imaging of specific regions, some alignment parameters (*R*^*ij*^ and **p**^*ij*^) were corrected to reduce the undesirable contribution of image artifacts to overall alignment. Pairings were considered unsatisfactory if they met at least one of the following criteria: (1) had a correlation coefficient lower than *R* = 0.5, (2) had an initial residual length larger than || **r** || = 300 pixels, (3) the resulting translation vector was larger than initial overlap dimensions, or (4) the resulting translation vectors would result in lack of overlap. The alignment parameters of these unsatisfactory pairings were corrected to *R* = 0.5 and **p** equal to an estimated translation vector. The estimated vector was calculated from the translation vectors with high-quality alignment, considering the type of overlap (see S1A and S1B Fig). High-quality alignments were defined as all pairs (*m*, *n*) that fulfill *R*^*mn*^ > 0.9 (S1C Fig). The estimated value of **p** of a specific alignment rejected by the aforementioned criteria was calculated as the geometric center of the set of vectors **p**^*mn*^.

To globally register the entire dataset, pairwise residual errors were minimized using mass-spring-damper (MSD) system dynamics similar to previously proposed elastic registration models [22,23]. We chose this approach as opposed to other more common approaches, such as least square (LS) minimization, as a way of reducing computer memory requirements while maintaining feasible computation times, which become an issue for large acquisitions such as the one presented here.

**Fig 2:**
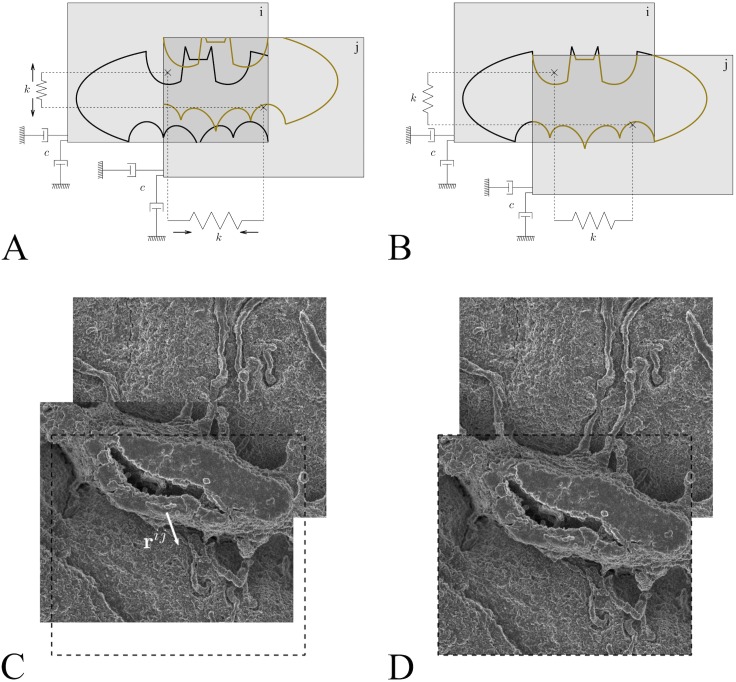
Global image registration was accomplished by modelling mass-spring-damper system dynamics. (A) Each image was assigned a point mass while each overlapping pair of images was connected via a spring. (B) An equilibrium spring length was defined at the translation that optimizes tile alignment, as calculated by phase-correlation. (C) Overlapping tiles and respective residual before alignment. (D) Aligned pair of overlapping tiles.

In a MATLAB simulation, each image tile was assigned a point mass concentrated at the image centroid, with the particles of each overlapping pair of images connected by a spring (Fig 2A). The springs were configured to exert zero restoring force, i.e. to reach their equilibrium length, if the corresponding pair of overlapping images was positioned in a way that maximizes pairwise alignment quality (Fig 2B). Image tiles (mass particles) have position, velocity and accumulated force at any given time instant.

The dynamics of the system, derived from Newton’s Second Law, with Hooke’s law and a damping force term, were modeled with the following second-order ODE:

$$m\overset{..}{x}+c\dot{x}+F_{k}=0,$$ (1)

where **x** is the tile position vector, the over-dot denotes the time derivative, *m* is the mass of each particle, *c* is the damping coefficient of particle motion, and **F**_***k***_ corresponds to the net spring forces vector. This term corresponds to the sum of the forces applied by all springs *j* connected to a tile *i*

$$F_{k}(i)=\sum_{j}\;\;k^{ij}\;\;r^{ij}$$ (2)

where

$$k^{ij}=k{\left(R^{ij}\right)}^{n}$$ (3)

is the spring stiffness and **r**^*ij*^ is the residual vector between tiles *i* and *j*. The string stiffness expression was defined as a power law of *R* to favor the contribution of regions with high quality images. Its expression is defined by the maximum spring constant *k*, weighted by the pairwise correlation coefficient *R*^*ij*^, and an arbitrary power *n*. Pairwise residual vectors (i.e. the relative positioning error between overlapping images) can be calculated using the position vectors of tiles (**x**^*i*^ and **x**^*j*^) and the relative image position vectors that best register the images as follows:

$$r^{ij}=(x^{i}-x^{j})-p^{ij}$$ (4)

The interconnected system of particles was configured with the mechanical parameters listed in Table 2, arranged with initial estimated coordinates recorded by the microscope stage, and allowed to come to equilibrium, reaching a lower energy configuration and maximizing alignment (S1, S2 and S3 Videos). The center tile of an arbitrary mFOV was assumed as an anchor point, and therefore its displacements in both directions for all time instants were considered zero. Differential equations were solved with MATLAB intrinsic function ode45, which implements an explicit Runge-Kutta method with a variable time step to perform time integration of the initial value problem. Optimal image tile montage was considered to be reached when the change in root-mean-square (RMS) of all residuals between consecutive iterations was less than 10^−6^ pixels. The integration step length was automatically chosen by the solver. All numerical simulations were calculated on an Apple Mac Pro with 3.5GHz (6 core) Intel Xeon E5 processor in single-thread mode and 64GB of memory, running OS X 10.11.1. Parametric studies (not included in this manuscript) found simulations with *n* = 5 to yield the lowest residual RMS and a damping ratio $\zeta =c/(2\sqrt{mk})\approx 0.14$ (m = 1 kg, *c* = 0.25 Ns/m) to converge to solution in shorter computational times.

**Table 2:** Parameters used for the mass-spring-damper system model.

*m*	1 kg
*k*	1 N/m
*c*	0.25 Ns/m
*n*	5

We compared our global optimization approach with both unweighted [21] and weighted least squares solutions using a collection of 60 mFOVs (3,660 tiles), excluding areas with widespread image artifacts. Least square solutions were calculated using the lsqnonlin function of Matlab, while the weighted optimization minimized transfer errors multiplied by *R*^*n*^ (*n* = 5, same weight function used in the stiffness of our springs).

### 2.5. Maps

Once fully registered, the stitched dataset was imported into TrakEM2. Variations in brightness amongst image tiles were minimized using non-linear blending [21].

Geographic information system (GIS) frameworks, such as Google Maps, frequently use pre-rendered, multi-resolution sets of images, referred to as a tiled pyramid structure. We adapted a TrakEM2-based CATMAID [23] exporter script [Beanshell script developed by Stephan Saalfield, https://github.com/axtimwalde/fiji-scripts/blob/master/TrakEM2/catmaid-export2.bsh] to render the tiled pyramid (S2 Fig) structure consisting of 11 zoom levels ranging from 0–10. The final, reconstructed mosaic of all single-beam images was partitioned in PNG-compressed 256 x 256 pixel tiles that collectively constitute the maximum zoom level (highest resolution). Tiles of higher zoom levels were recursively rendered by grouping 'squares' of four tiles (512 x 512 pixels) and downsampling each 'square' to a single, low-resolution 256 x 256 tile, doubling pixel size for each unitary decrement in zoom level.

Using the Javascript application programming interface (API) of Google Maps, we created a custom map of a human femoral neck region, which was made freely accessible to the public on www.mechbio.org/ploscompbiol. The pre-rendered pyramid tile directories were uploaded to a web server with unique directory paths: '(maxzoom—zoom)/y/y\_x\_(maxzoom—zoom).png', where maxzoom represents the maximum zoom level, and x and y are the positions within the tile coordinate system, as specified in the Google Maps API custom map documentation [https://developers.google.com/maps/documentation/javascript/maptypes]. The MapType interface was utilized to create custom maps and specify the translation from screen to tile coordinate frames.

## Results

### Phase-Correlation

**Fig 3:**
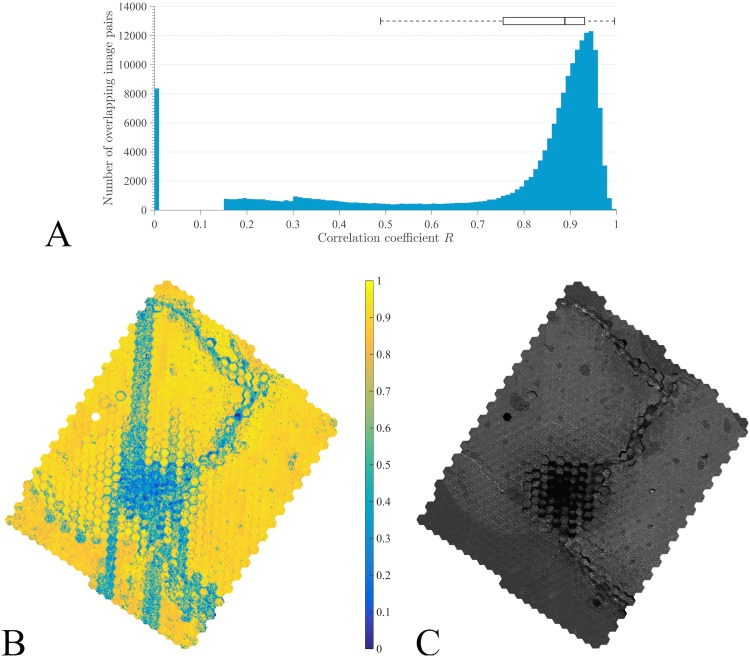
Phase-correlation coefficient, *R*, calculated for all image tile alignments. (A) Histogram showing distribution of *R* values. In the boxplot, the median is shown as a black line, and the box contains 25th and 75th percentiles of the coefficient data. (B) Spatial distribution of phase-correlation coefficients, with low *R* values contained within well-defined areas. (C) Reconstructed dataset following stitching. The dark areas in the overview image are attributed to deep surface depressions, likely originating from the combination of residual stresses in bone and the sample preparation process, which in turn compromise secondary electron detection. These correlate with low *R* values in B.

The histogram distribution of correlation coefficient values, calculated for more than 180k pairs of image overlaps, was negatively skewed, with overlapping image pairs concentrated at high correlation values (close to 28% of all pairwise correlations were below *R* = 0.8), and a median *R* value of 0.89 (Fig 3A). Overlapping image pairs with low *R* values were spatially concentrated in specific regions (Fig 3B) that correspond to the presence of local imaging artifacts observed in the reconstructed dataset after stitching (Fig 3C). Dark regions with disconnected signal patches were evident in the center of the scanned area and along the crack progressing diagonally upwards. These imaging artifacts are manifested as nonexistent, low, or blurred signals (S3 Fig) and were attributed to concavities of specimen topography that shielded the secondary electrons, generated in those regions, from the detector. Blurred areas, specifically, correspond to the regions with low *R*, arranged as vertical columns in Fig 3B.

### Global Registration using MSD vs Least Squares

**Fig 4:**
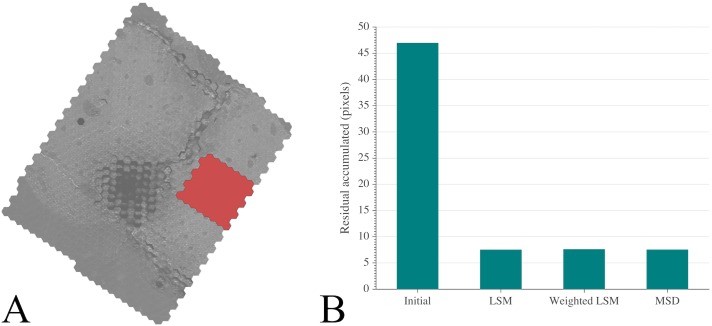
Comparing global registration using a mass-spring-damper dynamics approach with least squares methods. (A) Region of 60 mFOVs excluding areas with widespread image artifacts, highlighted in red, where stitching was computed. (B) Root-mean-squares of the residuals for initial configuration and 3 different global optimized registration approaches: unweighted least squares method (LSM), weighted LSM and MSD. Overall similar alignment error reductions were obtained. Registration with our mechanical model reduces RSM of residuals slightly more than the other approaches.

All three approaches (unweighted and weighted least squares minimization and our relaxation model), calculated for a region of 60 mFOVs (Fig 4A), showed similar ability to minimize registration errors, reducing the RMS of residuals by nearly 84% (Fig 4B). Least squares approaches (unweighted and weighted) converged within slightly shorter CPU times (~48 minutes) than our MSD approach (~54 minutes). However, LS approaches required over 10 times more RAM than our MSD approach (7270 MB and 710 MB respectively).

### Residuals

**Fig 5:**
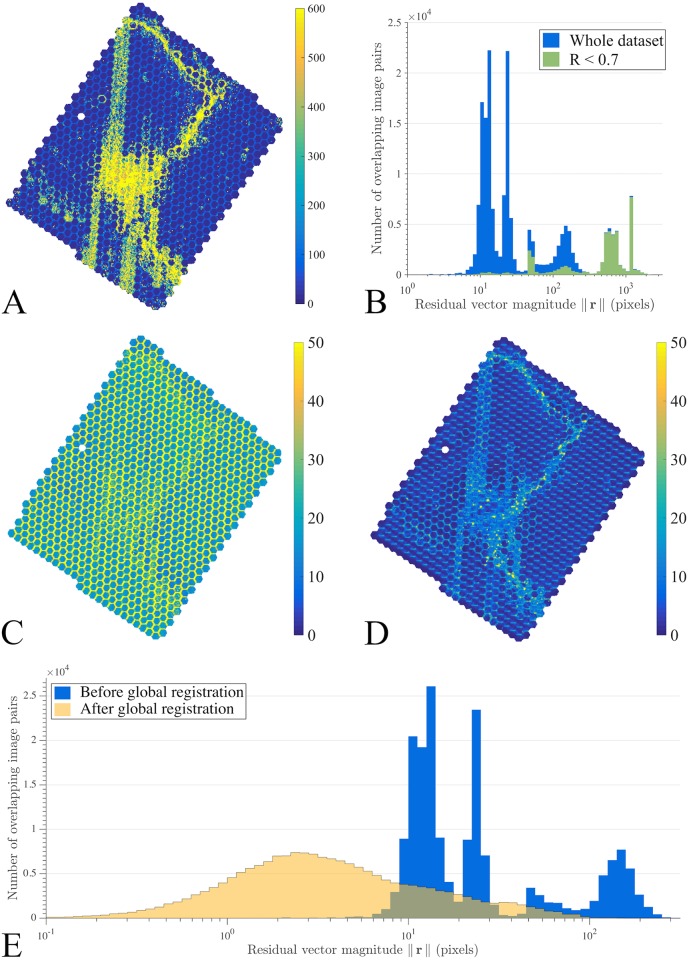
Stitching quality. (A) Map of local residual vector magnitudes, || **r** ||, calculated prior to alignment parameter correction. Large residual lengths correlate with regions having low *R* values and imaging artifact presence. (B) Histogram of the distribution of residual vector magnitude on a log scale for whole dataset (0 < *R* < 1) and for pairings with *R* < 0.7. Lower quality pairings have residual magnitudes concentrated within the interval || **r** || > 300 pixels. (C) Local || **r** || values after correction of alignment parameters (*R* and || **r** ||) and before global registration. (D) Local || **r** || values following stitching. (E) Histogram of residual magnitudes, || **r** ||, before and after global registration.

In the complete dataset, consisting of 897 mFOVs (54,717 tiles), the length of residual vectors, || **r** ||, was inversely related to local *R* values. A clear relationship exists between the spatial distribution patterns of || **r** ||, calculated before alignment parameter correction described in Section 2.4 (Fig 5A), and local correlation coefficients, *R*, shown in Fig 3A. Areas of large residual vector lengths were associated with regions that have poor pairwise alignment. Low *R* calculations can therefore lead to singularities on the large translation estimates, which are not representative of actual alignment corrections. The vast majority (97.3%) of residual vector lengths greater than 300 pixels correspond to pairings with correlation coefficient *R* < 0.7 (Fig 5B). This motivates the correction employed in translation parameters and the expression used for spring stiffness in Eq 3, which guarantees lower forces applied in tile pairs with low coefficients (and in most cases very large, nonrepresentative residuals) when compared to regions with high alignment quality.

Our mechanical system-based optimization algorithm was able to reduce the RMS of residuals by 76.6% and local || **r** || values, on average, by 72.6%. Fig 5C and 5D, respectively, show the spatial arrangement of || **r** || prior to and following stitching. Although there was an overall reduction in residual lengths, Fig 5D shows an increase in residual magnitudes across these artifact regions, in response to a lower stiffness of modeled springs assigned in these low *R* regions. Residual lengths, calculated after correcting alignment parameters prior to (C) and following stitching (D), shifted on average from 42 to 8 pixels and overall RMS decreased from 47 to 11 pixels (Fig 5E). Image pairs within the same mFOV had the lowest residual error, while the larger residuals were concentrated in regions of low *R*, as our algorithm favors alignment in regions with a high *R*. Global registration used around 1GB of RAM memory.

### Map

**Fig 6:**
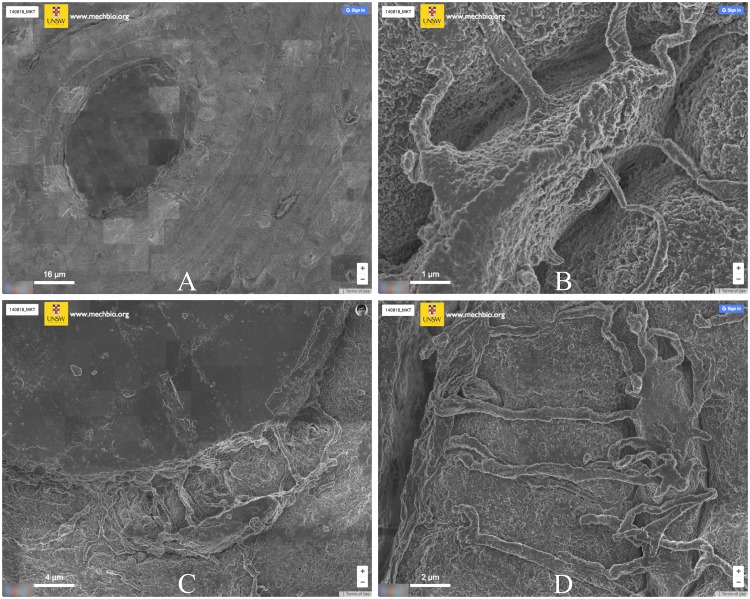
Screen captures from the reconstructed dataset, following stitching (www.mechbio.org/ploscompbiol). (A) Osteons, the functional units of cortical bone, are clearly visible with central blood vessels surrounded by layers of lamellar bone. (B) A close-up of an osteon. (C) An osteocyte adjacent to a blood vessel. (D) An osteocyte with processes adjacent to a crack.

The reconstructed maps reveal a detailed axial view of the composite nature of human cortical bone (Fig 6). Osteonal structural features are clearly visible with various bundles of blood vessels surrounded by lamellar bone, forming Haversian systems, distributed throughout the cross-section. Acid-etching these samples helps reveal the biological population of the tissue. Bone cells protrude from the mineralized matrix, revealing the morphological characteristics of osteocytes embedded within the mineral bone.

## Discussion

Recent advances in multi-beam SEM enable continuous nanoscale resolution imaging of macroscopic tissue samples and an overall increase in throughput greater than one order of magnitude compared to single beam electron microscopy. Here, we present computational methods to reconstruct a multitude of high-resolution images and develop interactive maps of mSEM datasets, allowing seamless navigation between length scales. The resulting maps reveal, with unprecedented detail across a range of length scales, the biological and material constituents and architecture of human bone. Below, we critically discuss the challenges of the presented methodology, with the goal of facilitating its routine use for structure-function characterization of biological tissues and their cellular inhabitants across length scales.

Quantitative assessment is critical for the evaluation of novel imaging techniques. Considering the association between local imaging artifacts and corresponding low pairwise image alignment score, we can assign the correlation coefficient *R* as an indicator of image quality. Successful (non-zero) calculations having an *R* = 0.89 median, suggesting that a simple 2D translational motion model with Fourier-based alignment is an adequate approach to register mSEM images. Overall, low interimage correlations were constrained to the regions of the sample with topographic depressions and cracks.

Surface anomalies reflected the challenge of preparing macroscopic hard tissue samples using methods designed for atomic force and electron microscopy, where residual stresses in macroscopic samples are released during sample preparation. Such topographical anomalies promote surface charging of electron beams and therefore compromise secondary electron detection, resulting in a low local signal-to-noise ratio. Low signal regions manifested as locally darkened and/or blurred areas in the raw images (S3 Fig). Blood vessels, due to their smoothness and resulting lack of contrast in acquired images, also hindered pairwise alignment and registered with a low *R*. Efforts are currently underway to further develop sample preparation methods that avoid formation of such anomalies.

Due to the extreme heterogeneity of properties amongst soft, organic inhabitant cells and their hard, inorganic composite environments [8], bone is one of the most challenging tissues to image across various length scales. Previous studies highlight the specific challenges of sample preparation with regard to etching of such bone composite specimens [15,17]. This was the first time bone was tested in such technical context. With increased precision and experience in sample preparation methodologies, however, such surface anomalies can be avoided [10]. Therefore, the presented results should be interpreted with consideration of our sample characteristics, recognizing that the presence of low-quality alignments is markedly induced by local sample conditions. Hence, in context of all tissues that make up the human body, bone provides a robust testbed for the technology.

Our results suggest that mass-spring-damper dynamics provide a rational and practical approach to perform global registration of mSEM acquisitions, which typically yield extremely large collections of tiles, reaching a solution at lower computational costs when compared to least squares minimization. Our MSD global registration algorithm corrected for intrinsic tracking discrepancies, requiring lower computational costs and similar final tile coordinates to LS approaches. For the 60 mFOVs highlighted in Fig 4A, the difference in final tile positioning between weighted LSM and the MSD model was on average 5.6 pixels. Yet, MSD outperformed LS in both computational time and memory usage.

Both LSM and MSD approaches converged to a similar residual error, suggesting that some distortions cannot be overcome with rigid translation models. Future studies will compare, under consideration of computational cost, the accuracy of direct (pixel-intensity matching) alignment against other more accurate registration approaches. Feature-based methods, for instance, apply feature extraction (e.g. MOPS [24], SIFT [25]) and global correspondence algorithms to estimate the geometric transformation model. These methods may apply a combination of translational and affine motion models to the dataset, which in the presence of sample-induced artifacts could improve pairwise registration in areas surrounding sample surface imperfections. Non-rigid models that account for aberrations induced by lens effects will also be taken into account in the alignment [26]. Additionally, we will also include a gain compensation step, to reduce intensity variation in overlapping regions, as seen in Fig 6A.

Our resulting map shows the composite multiscale architecture of bone, revealing both its structural intricacies and biological *milieu*. This reconstructed dataset, composed of more than 54,000 megapixels, has a considerably large field of view of 5.7 mm^2^, which compares to some of the largest nanometer resolution, electron microscopy fields of view in current literature [27]. Remarkably, the imaging of our specimen was performed in a practical amount of time, *i*.*e*. just under 3.5 hours. Furthermore, with the potential to scale mSEM technology according to higher beam counts, timeframe and throughput limitations will eventually become even less of a factor [28].

This combination of high throughput microscopy and image reconstruction with an online geographic information system (GIS) tool provides unparalleled, worldwide accessibility of human tissue images to the scientific community and public alike. The Google Maps platform, specifically, is a well-supported, familiar, user-friendly framework that allows for basic navigation of our dataset. Future developments will aim to share our maps in CATMAID [29], which will allow for collaborative annotation and bookmarking of regions of interest.

Top-down and bottom-up approaches look to explore the connectivity of biological and cellular components within certain physiological systems, to further model and analyze the progression of disease throughout the body [4]. The ability to image sub-cellular to tissue-organ scale structures is critical in providing an integrated understanding of physiological mechanisms [2,30,31]. A variety of different imaging modalities are usually required to bridge the gaps amongst various length scales.

Nonetheless, when coupled with the image reconstruction method presented in this paper, multi-beam SEM enables high-resolution characterization of biological tissues across a wide range of length scales. Aside from the modeling and analysis of tissues and organs, biocompatibility at the interface of an implant can also be assessed. Bridging local (*e*.*g*., how bone cells adapt extracellular matrix to optimize structure for dynamic function) to systemic perspectives, our method enables comprehensive characterization of multiscale biological phenomena including tissues, cells, and molecules. Such a step forward can be used in health diagnostics and to study health and disease etiology, enabling one to understand how tissue viability and cell connectivity relate to the disruption and failure of tissue and to the pathogenesis of diseases in organs. Already, a few recent studies in connectomics have utilized mSEM to render volumetric image data from murine specimens sectioned with a microtome and reconstruct neuronal circuits with single-synapse resolution [14]. Multiscale imaging of interfaces between musculoskeletal tissue compartments could reveal precise architectures of tight junctions that control functional barrier properties, which exert profound effects on human physiology [1,2,12,32].

In conclusion, our work provides the methodology to create large high-resolution images of biological tissues for structure-function characterization. Combining mSEM methodology with efficient stitching algorithms and GIS applications enables efficient navigation and dissemination of large collections of image data, as shown in this study, delivering a practical approach to assess materials over a wide range of scales. Open access of our reconstructed mSEM datasets, via the Google Maps platform, provides unparalleled, world-wide accessibility of human tissue images to the scientific community and public alike, in a well-supported, familiar, and user-friendly framework. This enabling step leads to a more complete understanding of health and disease, from the length scale of a single cell to the complex system of the human body.

## Supporting Information

S1 Fig Estimating translation vector p for image pairs with unsatisfactory alignment. (A) All possible types of image overlaps for a tile *t*. Overlap types 1 and 2 represent adjacent tiles from the same mFOV. (B) Corresponding relative coordinates prior to stitching, **x**^*i*^—**x**^*j*^, for all image pairs *i*, *j*. (C) Calculated 2D registration alignment vectors, **p**^*mn*^, for tile pairs *m*,*n* with *R*^*mn*^ > 0.9. The estimated translation vector **p**^*ij*^ was defined as the geometrical center of each group.
(TIF)

S2 Fig Tiled pyramid structure diagram. Tiles of higher zoom levels were recursively rendered by grouping 'squares' of four tiles (512 x 512 pixels) and downsampling each 'square' to a single, low-resolution 256 x 256 tile, increasing pixel size each unitary decrement in zoom level by a factor of 2.
(TIF)

S3 Fig Examples of imaging artifacts shown in the reconstructed mosaic. (A) Dark region caused by topographic depressions. (B) Close-up detail of white box in (A). (C) Detail of artifacts caused by crack in sample. (D) Region with high quality and adjacent blurred tiles.
(TIF)

S1 Video Diagram of relaxation algorithm in five hexagonal fields of view. Lines represent springs. Line color represents the respective local residual magnitude (green and red corresponding respectively to low and high residual lengths).
(AVI)

S2 Video Animation of relaxation algorithm in five hexagonal fields of view, for tiles corresponding to those depicted in S1 Video. (AVI)

S3 Video Magnified view of single osteocyte from S2 Video. (AVI)
