# Supplementary figures and images for: Creating High-Resolution Multiscale Maps of Human Tissue Using Multi-beam SEM
Source: PLoS Comput Biol. 2016 Nov 21;12(11):e1005217. doi: 10.1371/journal.pcbi.1005217 (PMC5117996; doi:10.1371/journal.pcbi.1005217)

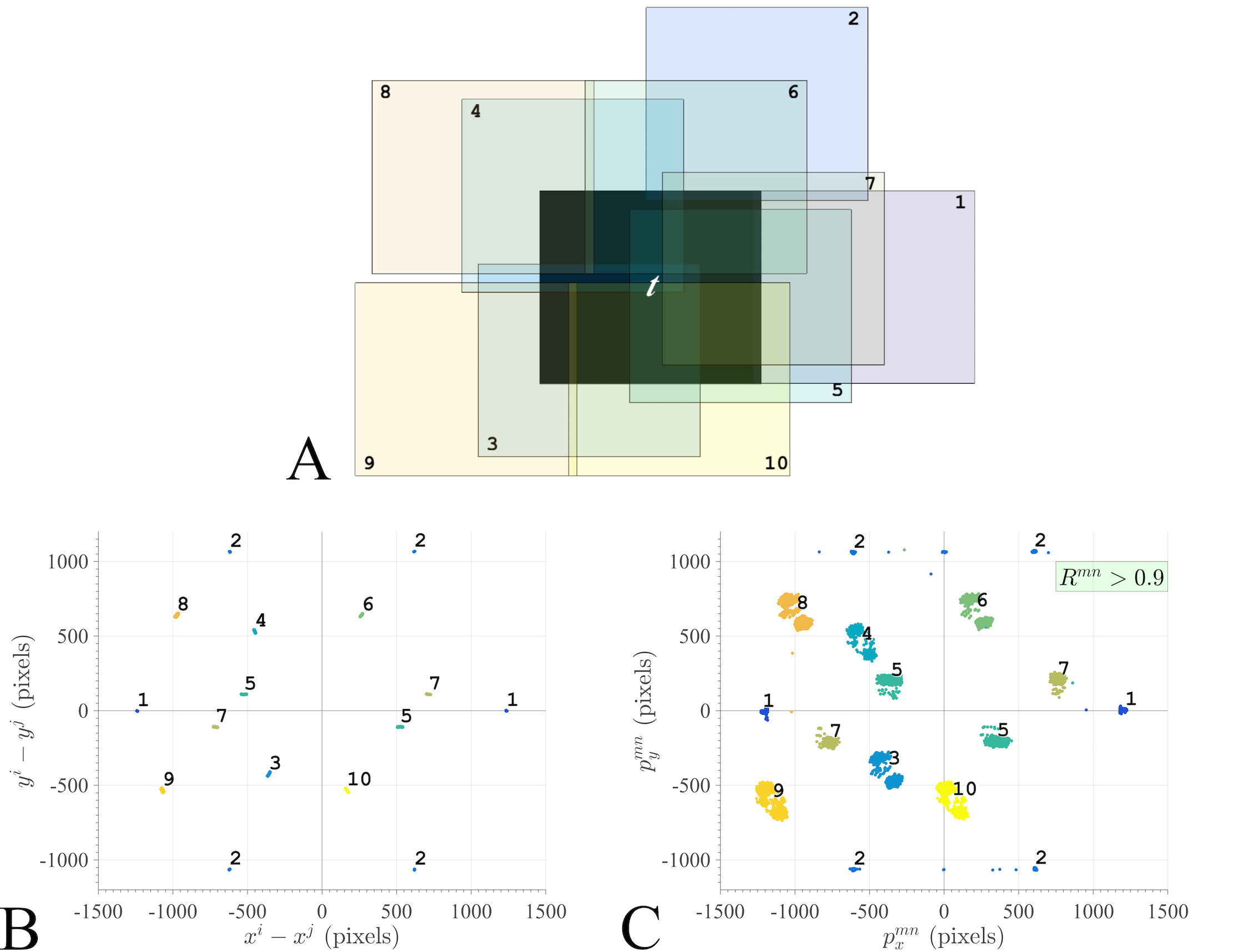

Supplement: S1 Fig — (A) All possible types of image overlaps for a tile t. Overlap types 1 and 2 represent adjacent tiles from the same mFOV. (B) Corresponding relative coordinates prior to stitching, xi—xj, for all image pairs i, j. (C) Calculated 2D registration alignment vectors, pmn, for tile pairs m,n with Rmn > 0.9. The estimated translation vector pij was defined as the geometrical center of each group. (TIF) [file pcbi.1005217.s001.tif]

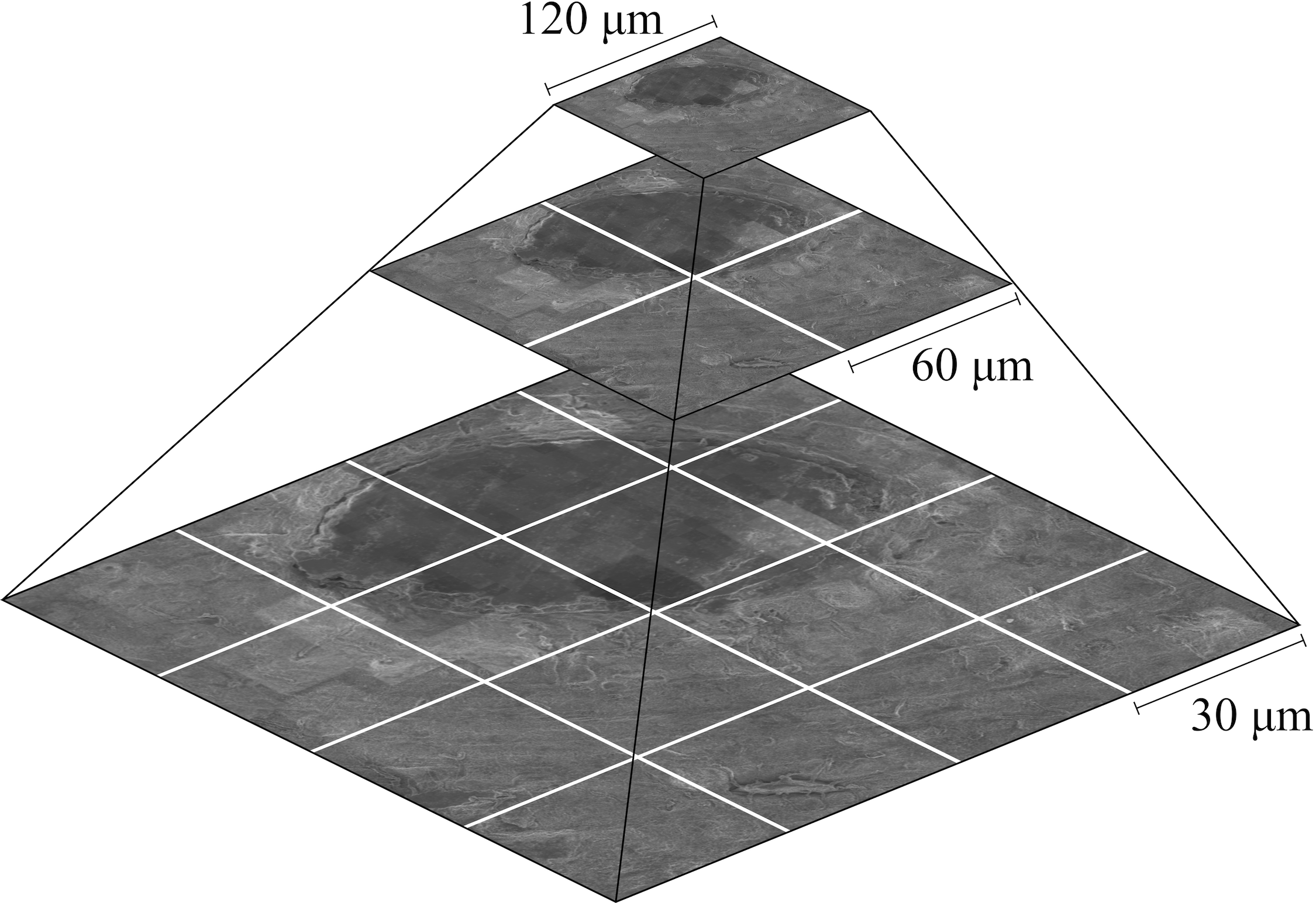

Supplement: S2 Fig — Tiles of higher zoom levels were recursively rendered by grouping 'squares' of four tiles (512 x 512 pixels) and downsampling each 'square' to a single, low-resolution 256 x 256 tile, increasing pixel size each unitary decrement in zoom level by a factor of 2. (TIF) [file pcbi.1005217.s002.tif]

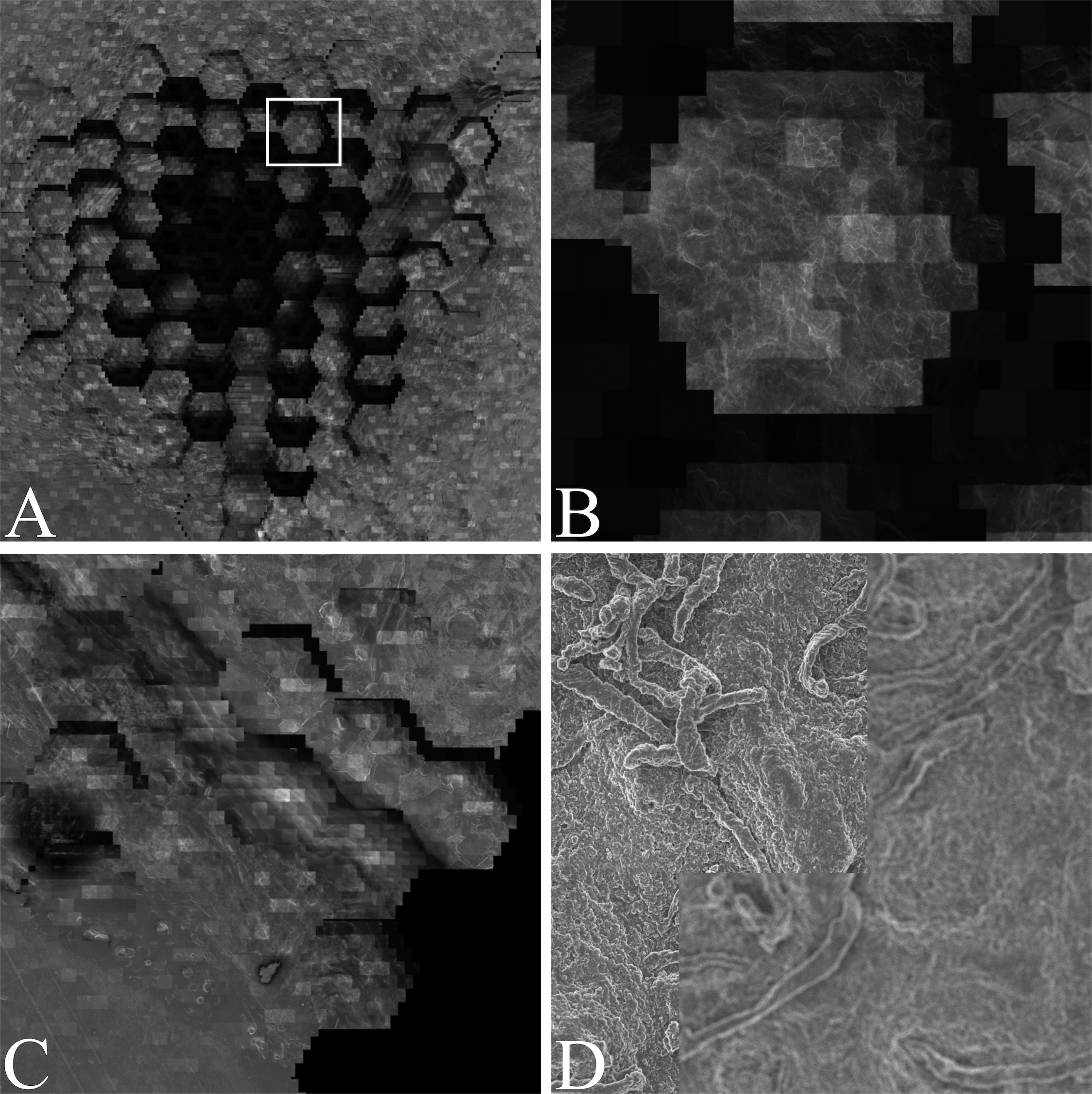

Supplement: S3 Fig — (A) Dark region caused by topographic depressions. (B) Close-up detail of white box in (A). (C) Detail of artifacts caused by crack in sample. (D) Region with high quality and adjacent blurred tiles. (TIF) [file pcbi.1005217.s003.tif]
